# Spectral subtraction denoising preprocessing block to improve P300-based brain-computer interfacing

**DOI:** 10.1186/1475-925X-13-36

**Published:** 2014-04-04

**Authors:** Mohammed J Alhaddad, Mahmoud I Kamel, Meena M Makary, Hani Hargas, Yasser M Kadah

**Affiliations:** 1 Computer Science, Faculty of Computing and Information Technology, King Abdulaziz University, Jeddah, Saudi Arabia; 2 Biomedical Engineering Department, Cairo University, Giza 12613, Egypt

**Keywords:** Brain-computer interface, Spectral subtraction, Wavelet shrinkage, Signal denoising

## Abstract

**Background:**

The signals acquired in brain-computer interface (BCI) experiments usually involve several complicated sampling, artifact and noise conditions. This mandated the use of several strategies as preprocessing to allow the extraction of meaningful components of the measured signals to be passed along to further processing steps. In spite of the success present preprocessing methods have to improve the reliability of BCI, there is still room for further improvement to boost the performance even more.

**Methods:**

A new preprocessing method for denoising P300-based brain-computer interface data that allows better performance with lower number of channels and blocks is presented. The new denoising technique is based on a modified version of the spectral subtraction denoising and works on each temporal signal channel independently thus offering seamless integration with existing preprocessing and allowing low channel counts to be used.

**Results:**

The new method is verified using experimental data and compared to the classification results of the same data without denoising and with denoising using present wavelet shrinkage based technique. Enhanced performance in different experiments as quantitatively assessed using classification block accuracy as well as bit rate estimates was confirmed.

**Conclusion:**

The new preprocessing method based on spectral subtraction denoising offer superior performance to existing methods and has potential for practical utility as a new standard preprocessing block in BCI signal processing.

## Introduction

Brain computer interfacing (BCI) is an important tool that allows direct reading of information from the subject’s brain activity by a computer. Such information can be used to perform actions controlled by the subject and hence provide an additional means of communication beside normal communication channels present in normal subjects. Such means can be the only way of communication with patients of such disease conditions as muscular dystrophy (MS) and therefore its development and enhancement have been the focus of many research groups in the past decade.

The brain activity at different locations can be measured using different methods that include electroencephalography (EEG), magnetoencephalography (MEG), and some functional imaging modalities such as functional magnetic resonance imaging (fMRI). These techniques offer brain activity signal time courses that come from a particular location in the brain with the resolution of such spatial localization ranging from a few signals for the whole brain (as with EEG) to signal for each 1 mm^3^ voxel within the subject’s brain (as with fMRI). The complexity of such systems also range from a simple, relatively inexpensive electrode cap worn by the subject and attached to a relatively small processing unit that provide very noisy signals while allowing subject mobility (as with EEG) to large expensive high field fMRI systems that allow excellent signal-to-noise ratio to be obtained while restricting the slightest subject motion during data acquisition. So, there is a clear trade-off between the quality of signals collected on one side and the mobility of the subject and the cost of the system on the other side. Approaches to improve quality of information from EEG-based systems through noise/artifact removal as well as more sophisticated analysis techniques would therefore allow this low cost, mobile technology to achieve better practical utility.

Several research articles addressed the problem of achieving higher quality of EEG signals for BCI applications and otherwise with aim to improve the signal-to-noise ratio (SNR).Two broad categories can be immediately recognized; namely, spatial domain techniques and temporal domain techniques. In the spatial domain techniques, the data from multiple spatially-distinct channels are utilized to identify the true signal projected onto all channels from the noise that is generally assumed to be independent among such channels. Such methods range from simple local spatial averaging to sophisticated variants of blind source separation methods such as independent component analysis [1-6]. On the other hand, temporal domain techniques attempt to find similarities within the time domain of a single channel signal that can be used to identify and suppress the noise components in that signal. This can be done by many methods ranging from simple averaging of consecutive epochs to transform domain based filtering techniques ranging from basic bandpass filtering [7,8] to different variants of the wavelet shrinkage method [9-18]. A hybrid method between spatial and temporal methods has also been recently proposed to take advantage of available channels and redundant signal epochs [19]. The predominant method of filtering used in BCI today is basic bandpass filtering that has become an essential part of the conventional preprocessing chain of BCI.

Even though previous denoising methods have contributed significant improvements, there are still limitations that need further research to reduce. For example, spatial domain methods rely on the availability of many channels (or electrodes), which would increase the cost, increase the weight, and cause loss of localization of EEG signals from the brain. Also, the integration of temporal domain signals into the preprocessing chain of BCI signals is yet to be done and is bound to increase the computational complexity requiring more expensive digital back-end hardware. Both techniques increase the power consumption of a portable BCI system due to additional channel front-ends or higher processing needed in the digital back-end. Therefore, a technique that would allow the use of a small set of channels and improve the performance of BCI system beyond the present methods at a reasonable computational cost would be highly desirable.

The aim of this work is to develop a denoising method for P300-based brain-computer interface data that allows better performance to be obtained with lower number of channels and blocks. The new method will be applied to experimental data and compared to the classification results of the same data using the same preprocessing and classification steps to allow direct comparison of results. Also, the new method will be compared to bandpass filtering and wavelet shrinkage based denoising as the relevant and widely used method for denoising at the present. Performance in different experiments will be quantitatively assessed using classification block accuracy as well as bit rate. The computational complexity of the new method is also described and compared to previous methods.

## Methods

The methodological approach that will be followed in this work is to adopt spectral subtraction based signal denoising, which is an effective speech signal denoising method that was previously applied to fMRI signal denoising [20]. This method uses adaptive estimation of noise and does not assume a model for the true signal thus matching well our problem. Here, we derive the spectral subtraction method for EEG applications and point out the modifications to the previous work to meet our unique application requirements.

Using the traditional additive noise model, the EEG temporal signal can be modeled as the summation of a true response signal, a physiological/instrumentation baseline fluctuation component, and a random noise component [20]. The physiological/instrumentation baseline fluctuation component can be considered as a deterministic yet unknown signal such as baseline drift or physiological motion artifacts and can be dealt with using existing preprocessing methods [21]. On the other hand, the random part consists of two components: the thermal noise in the electronics of the data acquisition system and the superimposed signals from neighboring neurons not involved in the true response sought. While the former component is well known to be Gaussian white noise process, the latter can also be shown to be so using a straightforward application of the central limit theorem to the summation of many signals of random activation patterns. Therefore, we will assume an additive noise model whereby the measured signal is practically the sum of a deterministic component *d(t),* including both the true EEG signal and low frequency or baseline wander, in addition to an independent random noise *n(t)*. That is,

(1) $$s\left(t\right)=d\left(t\right)+n\left(t\right).$$

Given that d(t) and n(t) are independent, the power spectrum of the measured signal can be given as,

(2) $$P_{\mathrm{ss}}\left(\omega \right)=P_{\mathrm{dd}}\left(\omega \right)+P_{\mathrm{nn}}\left(\omega \right).$$

Hence, the power spectrum of the deterministic part of the signal can be theoretically computed as [20],

(3) $$P_{\mathrm{dd}}\left(\omega \right)=P_{\mathrm{ss}}\left(\omega \right)-P_{\mathrm{nn}}\left(\omega \right).$$

So, the deterministic signal power spectrum is obtained by subtracting the spectra of the measured signal and an estimate of the random noise power spectrum. Practically speaking, to estimate that deterministic signal itself from the above estimated power spectrum, the magnitude of its frequency domain can be directly computed as the square root of the power spectrum. However, we need to find the phase part as well in order to be able to inverse-transform the frequency-domain estimate back to the time-domain signal. Several techniques can be used to do that. One such method relies on an estimate obtained from the phase of the Fourier transform of the original signal *S(ω)*[20]. In this case, the spectrum of the estimated deterministic signal *S*_*d*_*(.)* can be given as [20],

(4) $$S_{d}\left(\omega \right)=\sqrt{P_{\mathrm{dd}}\left(\omega \right)}\;\exp\left(j\;\mathrm{Phase}\left(S\left(\omega \right)\right)\right)$$

**Figure 1:**
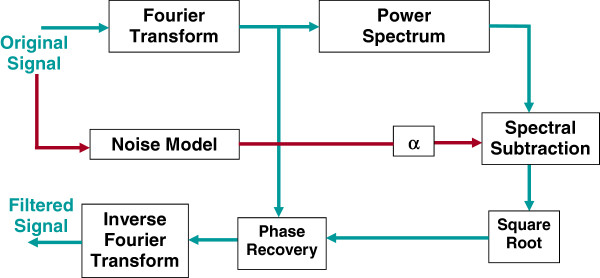
**Original Spectral Subtraction denoising block diagram.** (Appear in Methods). Original Spectral Subtraction denoising block diagram. The same data is used to estimate the noise power spectrum which is then removed from the overall power spectrum.

The denoised deterministic signal *s*_*d*_*(t)* is then computed as the real part of the inverse Fourier transformation of this expression. A block diagram of this method is shown in Figure 1.

**Figure 2:**
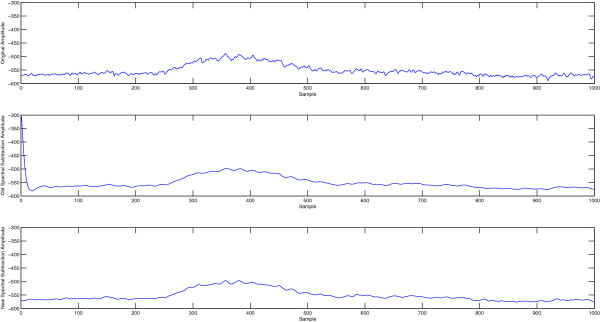
**Illustration of border discontinuity artifact in the old spectral subtraction method and its solution in the new method.** (Appear in Methods). Illustration of border discontinuity artifact in the old spectral subtraction method and its solution in the new method. The top plot shows the original signal, the middle plot shows the signal processed with the old spectral subtraction method with a clear artifact at the border in the beginning of the signal. This problem is absent in the new spectral subtraction method at the bottom.

In spite of the success of this method in denoising event related functional magnetic resonance imaging time courses, two problematic issues are present in our application to EEG recordings. The first is the use of the phase component of the original signal in the denoised signal. Given that the information in the phase part is as important as that in the magnitude part, leaving this component intact will no doubt limit the efficiency of the process in removing the noise components in the final signal. This issue was also a concern in the original application of this technique in fMRI and it was found that the improvement is still robust and therefore this issue is not as critical. The second issue is related to the observed jumps between the initial and final time points in the EEG epochs due to such effects as baseline drift that was found to be present and in many cases severe in the data sets we used in this work and elsewhere. This is an important difference between our case and the application of this method to fMRI signals where baseline wander is present but much less severe. Such large differences between first and last points in EEG epochs introduce incorrect high frequency components in the estimated power spectrum as a direct result of the discrete Fourier transform (DFT) model. The DFT assumes the measured epoch to be one period of a periodic signal, which means that the transform will see sharp discontinuities at both borders of the signal. As a result, this causes artifacts in the denoised signals that are deterministic yet unknown depending on the magnitude of such variable jump. This makes this technique not acceptable as a valid preprocessing tool in this application because of its introduction to such systematic errors. An illustration of such artifact is given in Figure 2 where the first part of a sample EEG epoch is shown before and after the old spectral subtraction processing. It can be observed that the beginning part of the denoised signal shows a clear artifact.

**Figure 3:**
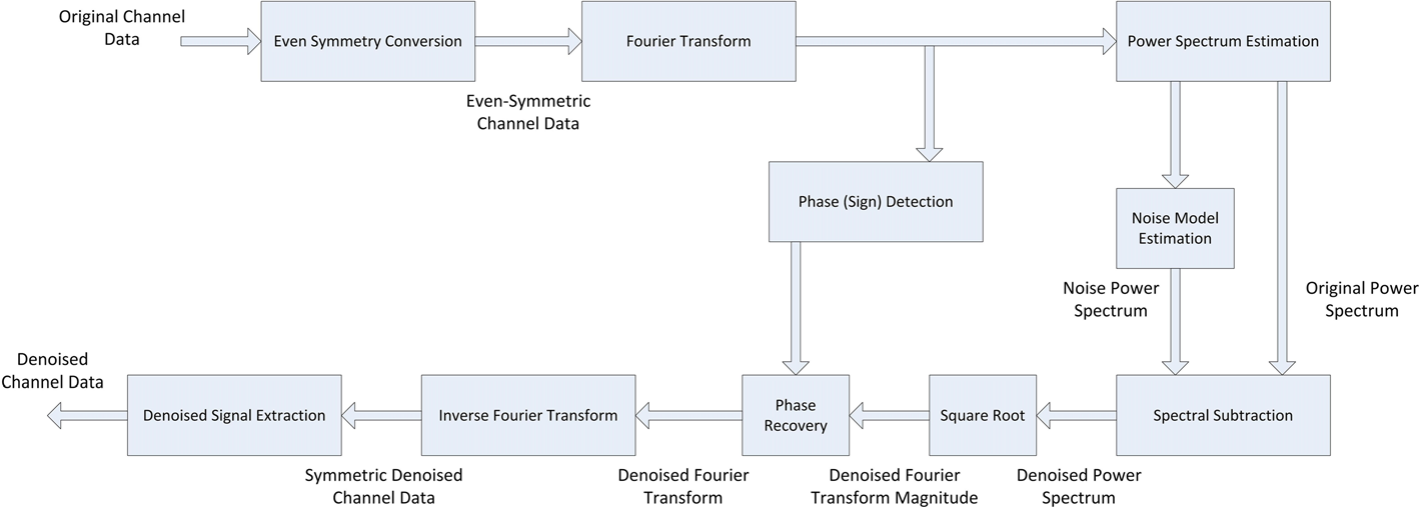
**Block diagram of Modified Spectral Subtraction Denoising.** (Appear in Methods). Block diagram of Modified Spectral Subtraction Denoising where the data are concatenated with its mirror image to generate a symmetric signal before applying the regular steps of the spectral subtraction method. This allows the phase of the signal to be zero and avoids artifacts from mismatch of signal levels at the borders.

To solve the above problem and allow artifact-free use of spectral subtraction, we here propose a modified version of the spectral subtraction method in which the original signal is converted to an even-symmetric signal by concatenating the signal with its time reversed version before using the discrete Fourier transform to estimate the power spectrum. This bears similarity to what is done in the widely-used discrete Cosine transform. This has two important implications that address the above two issues in the original method. First, the phase of this even-symmetric signal is expected to be zero for positive frequency amplitudes or π for negative ones. However, we observe a deterministic linear phase corresponding to a shift of ½ point since the origin of symmetry of this signal lies in between the two middle points. This changes the role of the phase estimation in the original method to merely sign detection and compensation for the deterministic ½ point shift yielding very high noise immunity. Second, the even symmetric signal form ensures the continuity at both ends of the signal to be preserved thus eliminating edge artifacts. The block diagram of the modified version of spectral subtraction is presented in Figure 3. The result of the using the modified spectral subtraction on the same signal in Figure 2 is shown at the bottom plot where the artifact present in the old spectral subtraction method is completely absent in the new method. The detailed steps of implementation of the new method are given as follows:

• Step 1: Read in the raw epoch data s(t) and convert it to a symmetric signal by concatenation with its reflected version s(-t).

• Step 2: Compute the fast Fourier transform of the symmetric raw epoch data. Estimate and keep the linear phase of the result.

• Step 3: Compute the periodogram-based estimate of the power spectrum as the squared magnitude of the fast Fourier transform of the raw epoch data.

• Step 4: Estimate the noise level by computing the average of the power spectrum values in the upper 20% of the frequency range that contains no signal components.

• Step 5: Use Equation (3) to compute the power spectrum of the denoised signal. If the subtraction result at any frequency is negative, it is clipped to zero.

• Step 6: Compute the denoised signal discrete Fourier transform as the square root of the denoised signal power spectrum and transform it back to the time-domain denoised signal after adding the deterministic linear phase estimated in Step 2.

## EEG signal noise power spectrum estimation

In order to implement the above denoising strategy, the noise power spectrum has to be estimated. Given that the noise model is Gaussian white noise, its power spectrum is well known to be constant over all frequencies that is directly proportional to the noise variance. Hence, it is sufficient to estimate a single parameter in order to completely determine the noise power spectrum.

**Figure 4:**
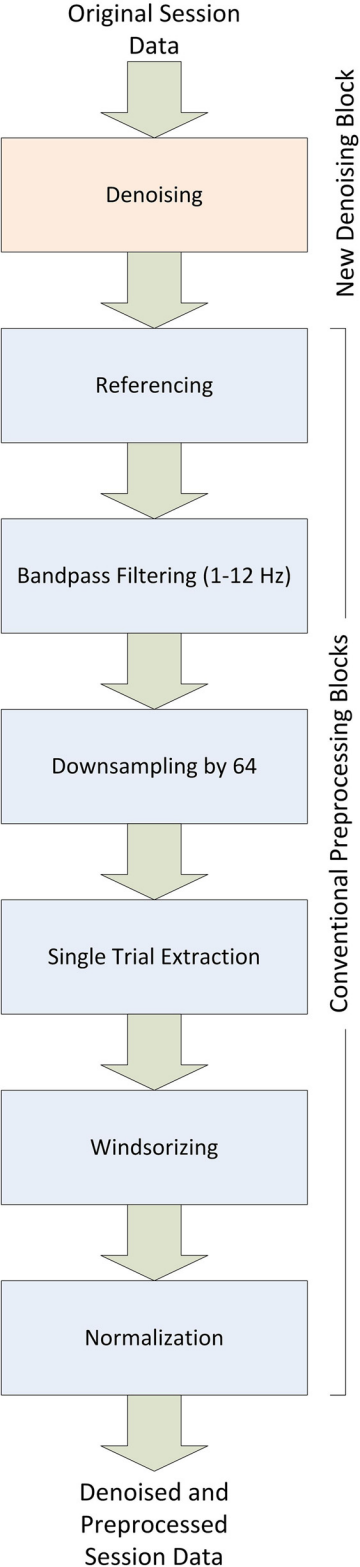
**Block diagram of proposed new preprocessing chain with an added new denoising block.** (Appear in EEG signal noise power spectrum estimation). Block diagram of proposed new preprocessing chain with an added new denoising block before the usual steps conventionally applied to BCI signals.

**Figure 5:**
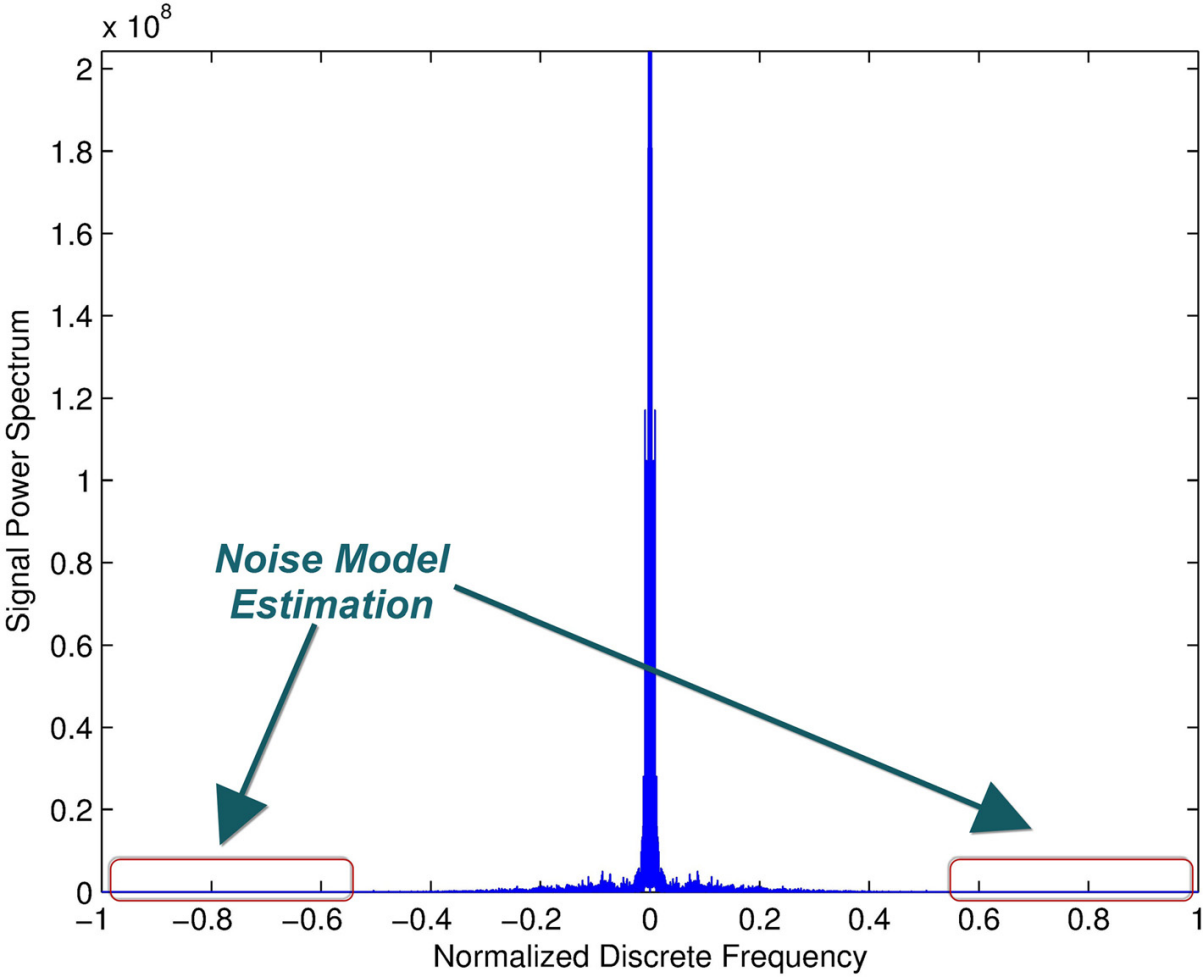
**Illustration of noise power spectrum estimation.** (Appear in Experimental verification). Illustration of noise power spectrum estimation from the upper part of the signal power spectrum on both ends known to have no true signal components based on the average of such areas.

Our strategy in this work is to have the new denoising technique implemented as a transparent block that can be used with existing trial extraction and preprocessing methods without any modifications to the other blocks. Therefore, we insert the new denoising block in between reading the session data file and the referencing step where the individual channel signals are read and processed using the new method then passed on to further processing steps in the same format they were read (as shown in Figure 4). Since this method should work adaptively, the estimation of the noise variance must be done adaptively from the original signals without any user intervention. This was done as follows. Since the original channel data are recorded using a much higher sampling rate than needed for the known frequency content of EEG signals and what is conventionally used for activation detection, the power spectrum of the original signal can be assumed to have noise only in its high frequency components. Consequently, the noise level can be estimated directly from the power spectrum of the original signal as the average of the upper half of the power spectrum as shown in Figure 5. The average is used because the power spectrum itself at each point can be shown to be a random variable that is unbiased (that is, mean is equal to true value) and consistent (variance decreases uniformly to zero as number of points goes to infinity). Given that the magnitudes of these points are independent and identically distributed, their average can be used to improve the estimation of the common mean of their processes. This estimation process is done for each channel independently and used to denoise its respective channel to account for different analog front-ends for each channel.

## Experimental verification

In this work, the data of Hoffmann *et al*. [21] were used to test the developed denoising method and compared it to both the case of no denoising and the case of wavelet shrinkage denoising [11,15]. We followed the exact same sequence of preprocessing and classification in this paper to allow the direct comparison between the two cases of preprocessing with and without the denoising step. The description of the data set is found in detail in [21] but a summary will be provided here. The duration of one run was approximately one minute and the duration of one session including setup of electrodes and short breaks between runs was approximately 30 min. One session comprised on average 810 trials, and the whole data for one subject consisted on average of 3240 trials. The impact of different electrode configurations and machine learning algorithms on classification accuracy was tested in an offline procedure. For each subject four-fold cross-validation was used to estimate average classification accuracy. The preprocessing operations applied were: referencing, bandpass filtering with cut-off frequencies set to 1.0 Hz and 12.0 Hz, downsampling by a factor of 64, single trials were extraction, windsorizing and finally amplitude normalization. The number of electrodes was selected as 4, 8, 16 or 32 depending on the experiment with the same electrode configurations in [21]. Then, the feature vector construction was done whereby the samples from the selected electrodes were concatenated into feature vectors. The dimensionality of the feature vectors was N_e_ × N_t_, where N_e_ denotes the number of electrodes (selected as 4, 8, 16, or 32) and N_t_ denotes the number of temporal samples in one trial (32 samples in our experiments). Classification of data was performed using Bayesian linear discriminant analysis (BLDA) and the software developed by [21] was used to perform this step. Given that the original signal passed through the standard preprocessing chain including the bandpass filter, comparing the results of different methods to it includes bandpass filter based denoising in the comparison. For the wavelet denoising, standard wavelet shrinkage denoising was used using Matlab with the basic wavelet chosen as “Coiflet-3” as suggested by [15] for direct comparison noting that we were able to get similar results using other basic wavelet functions (e.g., Daubechies-8). The universal threshold was selected with no multiplicative threshold rescaling [15].

## Results and discussion

**Figure 6:**
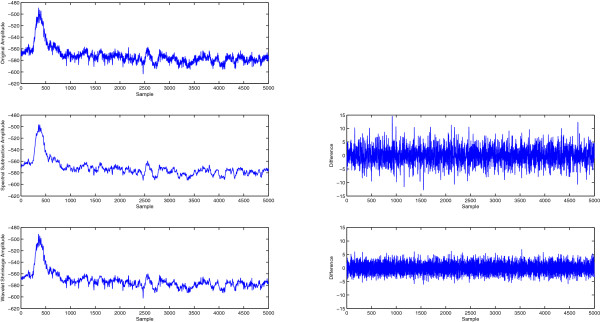
**Illustration of the results of spectral subtraction denoising as compared to the original signal and wavelet shrinkage denoising.** (Appear in Results and discussion). Illustration of the results of spectral subtraction denoising as compared to the original signal and wavelet shrinkage denoising. The left column shows from the top down: original signal, denoised signal using spectral subtraction, and denoised signal using wavelet denoising. On the right, the difference between the denoised signals and the original are shown to illustrate their random nature and show the relative amount removed between the spectral subtraction and wavelet methods. The amount removed by spectral subtraction seems to be higher than wavelet denoising for the same signal.

**Figure 7:**
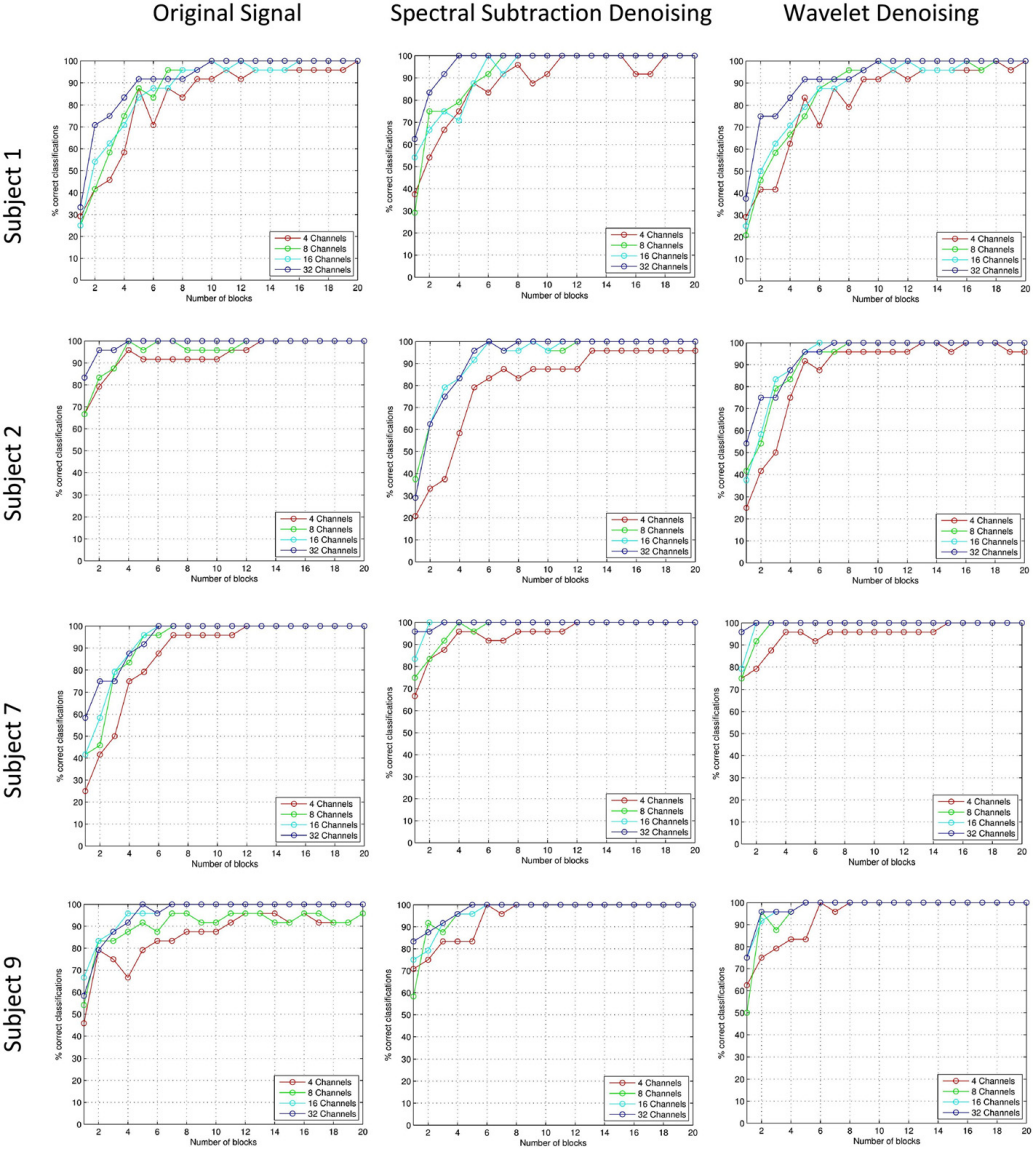
**Block accuracy results for different denoising methods vs. the original signal for sample cases.** Block accuracy results for different cases representing original signal (no denoising), spectral subtraction denoising, and wavelet shrinkage denoising for subject 1, 2, 7 and 9. The horizontal axis represents the number of blocks used to estimate the selection while the vertical axis represents the block accuracy reached.

**Figure 8:**
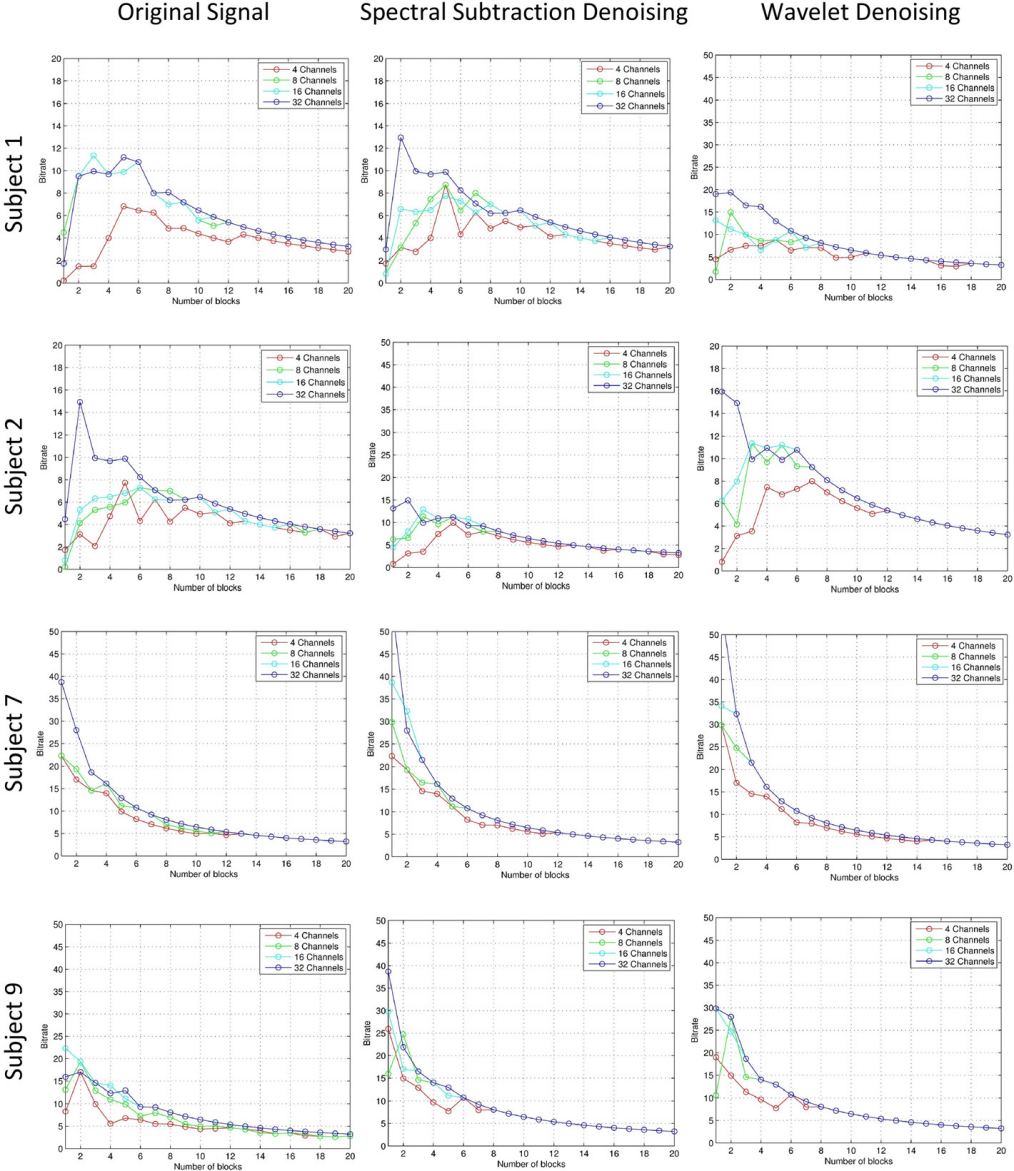
**Bit rate results for different denoising methods vs. the original signal for sample cases.** Bit rate results for different cases representing original signal (no denoising), spectral subtraction denoising, and wavelet shrinkage denoising for subject 1, 2, 7 and 9. The horizontal axis represents the number of blocks used to estimate the selection while the vertical axis represents the bit rate reached.

As an illustration to the denoising process, Figure 6 shows a segment of the original data and its spectral subtraction and wavelet denoising results. The left column shows from the top down: original signal, denoised signal using spectral subtraction, and denoised signal using wavelet denoising. On the right, the difference between the denoised signals and the original are shown to illustrate their random nature and having no signal components and show the relative amount removed between the spectral subtraction and wavelet methods. The amount removed by spectral subtraction seems to be higher than wavelet denoising for the same signal. The classification results of using the new denoising method are shown in Figures 7 and 8 where each figure consists of the results with bandpass denoising (original signal), results with spectral subtraction denoising, and results with wavelet shrinkage denoising. The results include data from 2 abnormal subjects and 2 normal subjects to illustrate its utility across different subject conditions [21]. The results are expressed as block accuracy graphs in Figure 7 and as bit rate graphs in Figure 8 for 4, 8, 16 and 32 channel data to study the impact of denoising under different data acquisition conditions using different measures [22]. The use of per-block accuracy rather than the per-trial accuracy is because the correct answer must be only one out of each block to unambiguously identify the selected target. Here, each block consists of 6 trials and therefore identifying more than one of them as selected would not provide a proper communication between the subject and the computer. The bit rate is a good indication to the effective communication channel bandwidth that measures the trade-off between the higher block accuracy achieved with more blocks and the time needed to collect them. Therefore, it is important to consider both metrics together to better interpret the results.

It can be observed that the block accuracy results for 4-channel data (plotted in red) show a significant improvement from the original data in both spectral subtraction and wavelet shrinkage methods with low number of blocks. This is also reflected as higher bitrates in the same range. Even though the effect of denoising in general is more apparent in experiments with lower number of channels and low number of blocks, there is still evident improvement in experiments with high number of channels where 100% accuracy is reached earlier as evident in all cases. This is important to indicate that the inherent spatial compounding from the many electrodes can still take advantage of temporal denoising methods and that a combination of the two yields the best results.

By inspecting the results further, we observe that the spectral subtraction method offers better results than wavelet shrinkage based denoising in most experiments with the exception of a few cases such as in the 4-channel data of Subject 2 where the 100% accuracy is maintained once reached in wavelet denoising while it does not with spectral subtraction. Nevertheless, in all other cases the spectral subtraction results are superior as evident in the achieved block accuracy and bit rate for any given experiment. As a general observation, the results of spectral subtraction and wavelet denoising methods show a clear advantage over the results with only bandpass filtering in the original signal. Since such denoising step can be inserted within the conventional preprocessing of BCI data, this study shows clear evidence that these more sophisticated denoising methods should be integrated as a standard step in the preprocessing chain to improve the SNR of the collected signals.

Assuming a data set of M channels with N points each, the computational complexity of spectral subtraction is ***O*** (M N log_2_ N). On the other hand, The computational complexity of wavelet shrinkage method varies with different implementation with a minimum complexity of ***O*** (M N^2^), which is significantly higher. For example, for N = 100,000 points and same number of channels, the wavelet shrinkage method will require N/log_2_(N) times the computations of spectral subtraction, which is more than 3 orders of magnitude higher. Therefore, the computational complexity of spectral subtraction is more efficient for applications requiring embedded implementations or fpr real-time processing.

The model used in data processing amounts to subtracting the noise component uniformly across all frequencies. This is different from conventional frequency selective filters that are equivalent to a convolution in the time domain that causes the noise components in different time points to be correlated in the output signal. Hence, a theoretical advantage of this method is its preservation of the independence of random components within the time points processed. Hence, it is well-suited for use with standard statistical analysis methods that require statistical independence of samples. An example of such methods is when improving statistical estimation by using data from multiple blocks where the presence of correlated rather than independent noise across blocks degrades the achievable improvement. Given that the wavelet shrinkage based methods involve frequency selective filters to compute its coefficients, the same advantage cannot be claimed for that method. This explains the overwhelmingly better performance of the spectral subtraction method than the wavelet shrinkage based method when the number of blocks is higher.

## Conclusions

In this work, a new denoising method for P300-based brain-computer interface data that allows better performance to be obtained with lower number of channels and blocks was developed. The new method was verified using experimental data and promising improved results were obtained. The new method was favorably compared to bandpass filtering and wavelet shrinkage based denoising as the present relevant and widely used method for denoising. Performance in different experiments using classification block accuracy as well as bit rate show significant improvement with a clear advantage in computational complexity. The results highlight the potential for including the new method as a standard preprocessing block for BCI data.

## Competing interests

The authors declare they have no competing interests.

## Authors’ contributions

MA is the *Primary Investigator* PI of the project under which this work was performed and contributed to the formulation of the idea and reviewed the manuscript. MK contributed to the formulation of the idea and the conduction and evaluation of experiments. MM contributed to the formulation of the idea and conduction of early experiments. YK contributed to the idea, was in charge of coding, conduction and evaluation of experiments, and drafted the manuscript and its revised versions. All authors read and approved the final manuscript.
